# Percutaneous mechanical thrombectomy in a patient with extensive deep vein thrombosis: pioneering surgery in the northern region

**DOI:** 10.1590/1677-5449.202501162

**Published:** 2026-03-23

**Authors:** Gabriel da Silva Nunes, Rayssa dos Anjos Cabral, Andressa Pereira de Souza, Leonardo Pappis Orso, Nelson Ogliari Rezende, João da Silva, William Wakasugui, Vinicius Tadeu Ramos da Silva Grillo

**Affiliations:** 1 Centro Universitário São Lucas – UNISL, Afya Porto Velho, Porto Velho, RO, Brasil.; 2 Instituto Vascular e Endovascular de Rondônia – IVER, Porto Velho, RO, Brasil.

**Keywords:** deep vein thrombosis, thrombectomy, endovascular procedures

## Abstract

This report presents the first documented case in North Brazil of percutaneous mechanical thrombectomy (PMT) performed in a young patient with extensive iliofemoral-popliteal deep vein thrombosis (DVT). The patient presented with pain and significant swelling of the left lower limb, with ultrasonographic evidence of extensive DVT. The ClotTriever® system was employed via a popliteal approach, combined with adjunctive embolectomy using a Fogarty catheter. The procedure was uneventful and led to rapid clinical recovery. At 14 months of follow-up, the patient was asymptomatic, and venous Doppler ultrasonography findings were normal. PMT proved to be safe, effective, and feasible as an innovative therapeutic option in a regional setting. This case underscores the technique’s potential role as a promising alternative for treatment of extensive DVT, particularly in centers outside major urban areas.

## INTRODUCTION

Deep venous thrombosis (DVT) occurs when thrombi form in veins of the deep vein system, is more common in veins of the lower limbs (80-95% of cases) and can cause complications such as postthrombotic syndrome (PTS) and pulmonary embolism (PE). Without appropriate treatment, 5 to 15% of patients with DVT will die from PE; while the combined incidence of DVT and PE is estimated at 2 cases per 1,000 people per year, with rates of recurrence as high as 25%.^1,2^ Moreover, the incidence of PTS in patients with DVT is in the range of 23 to 60% of cases and is more frequent in the 2 years following the DVT episode.^3^

Percutaneous mechanical thrombectomy (PMT) is an innovative strategy for DVT management that is capable of thrombus removal in an effective and minimally invasive manner without the need for use of thrombolytics. This report describes the first case in which PMT was performed in the North region of Brasil, demonstrating its applicability, efficacy, and safety.

This study was assessed and approved by the Research Ethics Committee (CAAE 85170324.0.0000.0013, opinion number 7.629.445).

## PART I – CLINICAL SITUATION

A 27-year-old male patient presented complaining of pain and paresthesia in the left lower limb (LLL) with onset 2 days previously. He reported systemic arterial hypertension and had undergone a gastric bypass 35 days previously. Laboratory tests showed leukocytosis with no shift, hemoglobin at 15.5 g/dL, hematocrit at 45.8%, prothrombin time of 45.8 seconds and elevated C-reactive protein (149.9 mg/L).

**Figure 1:**
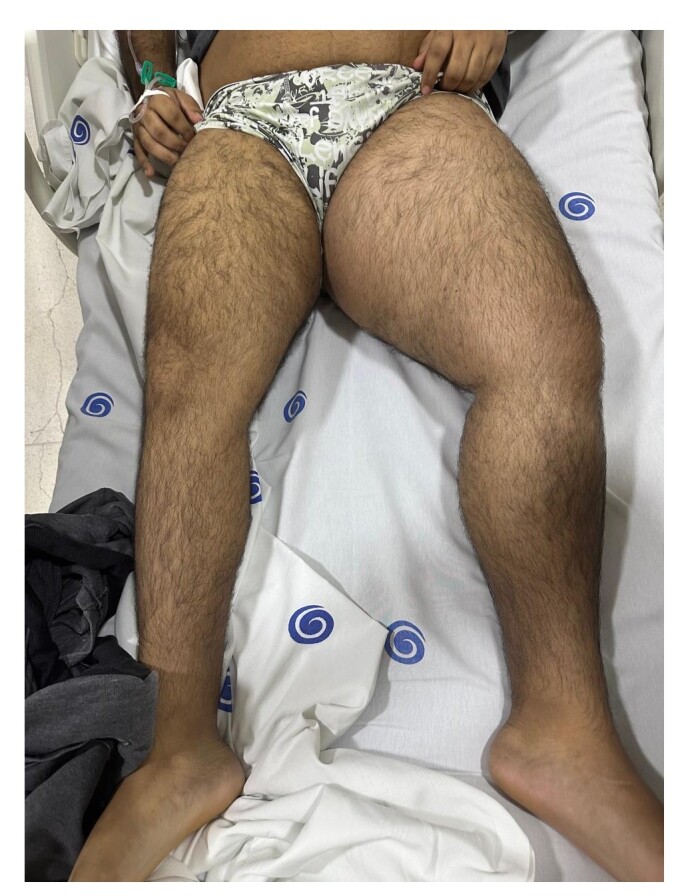
Patient with extensive asymmetrical edema and clubbing of the left lower limb (LLL), compatible with deep venous thrombosis (DVT).

On physical examination, the patient was stable, but with persistent pain, asymmetrical edema, clubbing, weak distal pulses, and peripheral cyanosis of the toes of the left foot (Figure 1). Venous Doppler ultrasonography detected DVT involving the external iliac, common femoral, deep femoral, femoral, popliteal, and medial gastrocnemius veins of the LLL.

## PART II – WHAT WAS DONE

**Figure 2:**
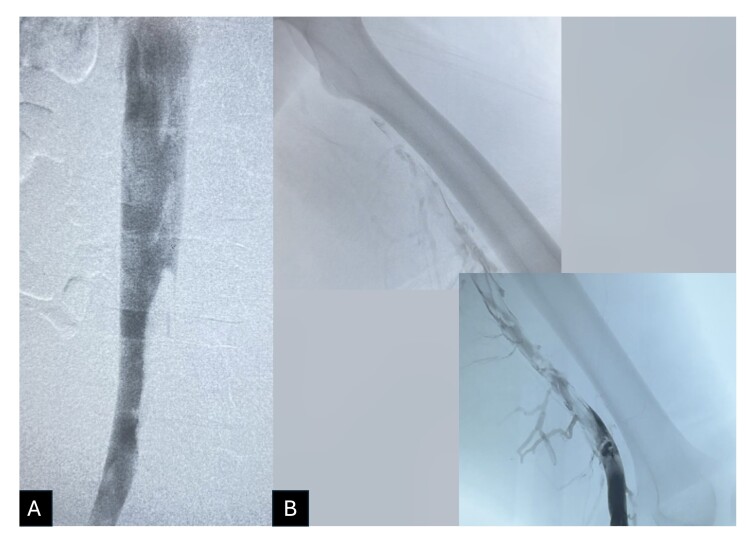
Phlebography conducted after ultrasound-guided puncture of the left popliteal vein and insertion of a 6Fr introducer. (A) Thrombus involving the left iliocaval segment; (B) Thrombus involving the left femoropopliteal segment.

The procedure chosen was PMT using the ClotTriever® system (Inari Medical, Inc., United States), treating the iliocaval and femoropopliteal segments of the LLL. The procedure was performed with local anesthesia and sedation. With the patient in the supine position and with the LLL flexed, the left popliteal vein was punctured with ultrasound guidance and a 6Fr introducer was inserted. Initial phlebography confirmed DVT from the popliteal vein to the inferior vena cava (Figure 2).

**Figure 3:**
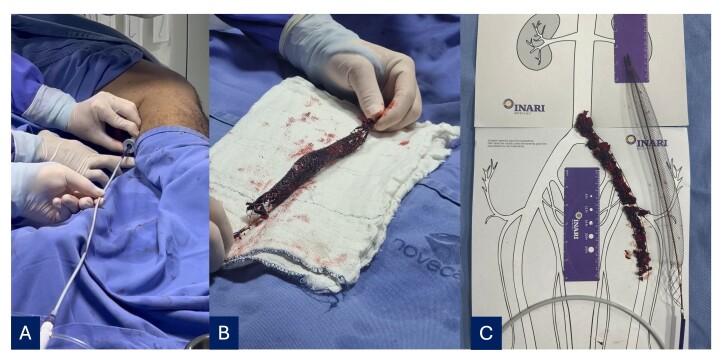
**(**A) Insertion of the catheter in the femoropopliteal region; (B) Extensive thrombus captured by the collection bag; (C) Length of the area involved and proportions of the collection bag*.*

**Figure 4:**
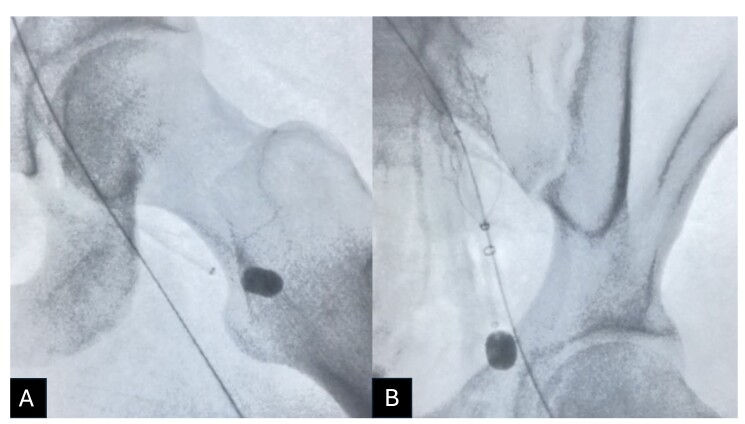
Radioscopy showing the procedure to remove thrombus from the left deep femoral vein. (A) Inserted via the right common femoral vein, the 6Fr x 55cm long introducer was positioned at the origin of the left deep femoral vein. The n° 4 Fogarty catheter was inserted via the long introducer, positioned in the distal segment of the left common femoral vein, and inflated with contrast diluted in saline; (B) The ClotTriever® system was positioned open in the left iliac segment and the introducer and Fogarty catheter were tractioned together up to the common femoral vein.

**Figure 5:**
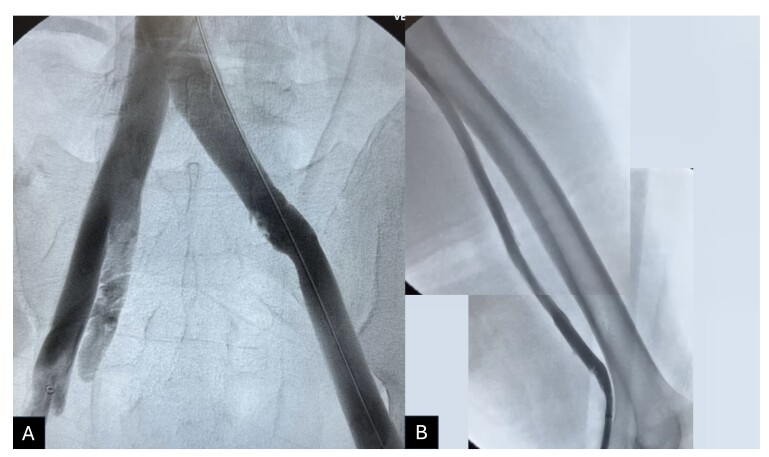
Showing phlebography conducted after thrombectomy. It is possible to observe that the treated veins are patent, indicating effective removal of the thrombi identified previously. (A) Iliocaval segment; (B) Femoropopliteal segment.

Percutaneous mechanical thrombectomy was performed using the ClotTriever® system, removing a large volume of recent thrombi (Figure 3). The right common femoral vein was punctured to access the deep femoral vein, the left deep femoral vein was catheterized, and a 6Fr x 55 cm long introducer was advanced. Embolectomy with a nº 4 Fogarty catheter enabled the thrombus to be directed towards the ClotTriever®, which had been placed in the left external iliac vein (Figure 4). Control phlebography showed that the treated segments were patent, free from residual thrombi, and had continuous blood flow (Figure 5).

**Figure 6:**
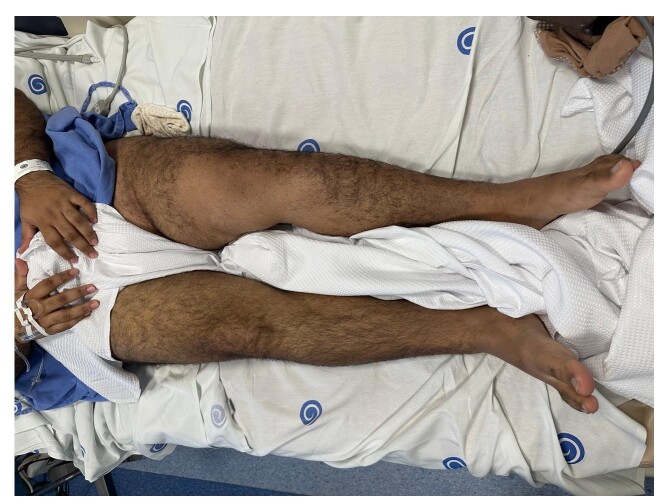
Photograph showing resolution of edema of the affected limb the day after surgery. The improved edema suggests venous flow has been reestablished, with no signs of postoperative complications, confirming the patient’s favorable recovery.

**Figure 7:**
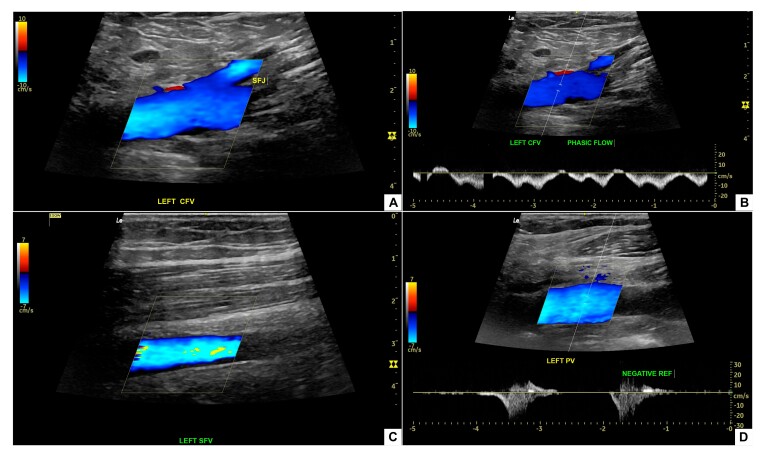
Vascular color and spectral Doppler performed 14 months after percutaneous mechanical thrombectomy. (A) Left common femoral vein, with preserved venous flow and complete recanalization; (B) Spectral Doppler of the left common femoral vein demonstrating respiratory phasic flow; (C) Left superficial femoral vein with maintained patency and no imaging findings compatible with residual thrombi; (D) Left popliteal vein with adequate flow and absence of venous reflux on spectral Doppler, confirming satisfactory hemodynamic recovery. ESQ = left; FLUXO FASICO = phasic flow; SFJ = saphenofemoral junction; NEGATIVE REF = negative reflux; CFV = common femoral vein; SFV = superficial femoral vein; PV = popliteal vein.

The patient was sent to the intensive care unit (ICU) and started on full anticoagulation with 100 mg enoxaparin every 12 hours, with elastic compression, the leg raised, and early mobilization. The next day, he exhibited considerable clinical improvement, with regression of the edema and no complications (Figure 6). A chest angiotomography showed no signs of PE. He was discharged after 1 day in the ICU and 2 days in a ward. At outpatient follow-up, 14 months after the procedure, he was asymptomatic and deep venous Doppler ultrasound findings were normal (Figure 7).

## DISCUSSION

Percutaneous mechanical thrombectomy constitutes an effective alternative to conventional treatment of DVT, especially in cases with extensive thrombosis, such as the case described. By avoiding use of thrombolytics, the risk of bleeding and other complications is reduced.^4^ Moreover, PMT is associated with a shorter length of hospital stay, with rapid clinical resolution, and with lower incidence of PTS.^5,6^

In the present case, the indication for percutaneous mechanical thrombectomy was driven by the extent of the DVT and the severity of the clinical presentation. The favorable outcome suggests the method is safe and effective, contributing to its validation as a treatment approach, especially at centers with a catheterization laboratory and endovascular expertise.^7^

In addition to its clinical efficacy, PMT also contributes to early return to normal activities, reducing the need for prolonged hospital stays and additional interventions.^8,9^ Seen through this lens, patients who undergo this procedure have faster functional recovery and are less likely to need additional intervention compared with conventional treatment, as was the case of the patient described in this report.

The literature shows that patients treated with PMT tend to have less need for ICUs and faster functional recovery, supporting the findings of the present study (Table 1). Introduction of this technique in the North region of Brasil is a significant development and could influence adoption of the procedure at other specialist centers.

**Table 1:** Publications in the literature demonstrating the positive results of PMT.

**Authors**	**Summary of results**
**Mouawad** **^5^**	Case report: successful single-session treatment of phlegmasia cerulea dolens with PMT, restoring venous patency and relieving symptoms.
**Nguyen et al.** **^9^**	Single-center cohort study: ambulatory management of iliofemoral DVT with May Thurner syndrome, using thrombectomy, angioplasty and stenting, with technical success and 86.7% patency after 12 months, compared with in-hospital management.
**Wong et al.** **^10^**	Systematic review: PMT for acute iliofemoral DVT had 75-100% patency rates, fewer hemorrhagic complications, and lower incidence of PTS. Moreover, rates of PTS and DVT recurrence were less than 17 and 15%, respectively.
**Maldonado et al.** **^11^**	Prospective observational study: the ClotTriever® system (Inari Medical, Inc., CA, USA) was effective for removal of thrombi in a single session, without the need for thrombolytics, with successful complete or near-complete removal in more than 80% of cases.
**Zia et al.** **^12^**	Retrospective single-center review: results demonstrated that PMT had similar reocclusion rates to other treatments, but with fewer hemorrhagic complications and without the need for an ICU stay.
**Abramowitz et al.** **^13^**	Randomized clinical trial: the study showed that mechanical thrombectomy with the ClotTriever® system is safe and effective for acute, subacute, and chronic DVTs, with significant improvements in quality of life and clinical symptoms, without notable differences between subsets.
**Crowner and Marston** **^14^**	Case report: a case of thrombectomy with the ClotTriever® for acute DVT demonstrated efficacy and safety for removal of thrombi without thrombolytics, resulting in clinical improvement at 6 months.

PMT = percutaneous mechanical thrombectomy; PTS = postthrombotic syndrome; DVT = deep venous thrombosis; ICU = intensive care unit.

Implementation of PMT for treatment of DVT in Brazil’s North region faces serious accessibility barriers because, in contrast to ischemic stroke, which is covered by Ministry of Health Ordinance number 5,^15^ guaranteeing coverage on the Unified Health System (SUS - Sistema Único de Saúde), there are no plans for widespread adoption of PMT for DVT, revealing health system inequalities.

The high cost of the procedure excludes patients who are dependent on the SUS or have insurance that does not cover high complexity treatment, forcing many to seek care in the private sector, with costs that are unfeasible for the majority of people. Despite the dimensions of the SUS, its limitations in offering advanced treatments such as PMT, compounded by a lack of clear protocols and the scarcity of resources in peripheral regions, exacerbates inequality, especially in the North of Brazil.

Adoption of PMT on the SUS is not merely justifiable in terms of the immediate clinical benefits, but also because of the overall savings, with reductions in chronic complications and inequality of access. It is therefore hoped that publication of this result encourages adoption of PMT at specialist centers, expanding access to innovative treatments and reducing the morbidity and mortality associated with DVT. Additional studies are needed to consolidate its incorporation into national treatment protocols.

## CONCLUSIONS

In the present case, PMT proved safe and effective for management of extensive DVT, with fast clinical recovery and absence of complications. This report is the first documented use of the technique in the North region of Brasil, highlighting its value for extending minimally invasive approaches in regional settings.

## Data Availability

Compartilhamento de dados não se aplica a este artigo, pois nenhum dado foi gerado ou analisado.
