# Supplementary material for: Percutaneous mechanical thrombectomy in a patient with extensive deep vein thrombosis: pioneering surgery in the northern region
Source: J Vasc Bras. 2026 Mar 23;25:e20250116. doi: 10.1590/1677-5449.202501162 (PMC13021007; doi:10.1590/1677-5449.202501162)
Supplement: Translation

# Translation: Trombectomia mecânica percutânea em paciente com trombose venosa profunda extensa: cirurgia pioneira na região norte

**DOI:** 10.1590/1677-5449.202501161

**Authors:** Gabriel da Silva Nunes, Rayssa dos Anjos Cabral, Andressa Pereira de Souza, Leonardo Pappis Orso, Nelson Ogliari Rezende, João da Silva, William Wakasugui, Vinicius Tadeu Ramos da Silva Grillo

**Affiliations:** 1 Centro Universitário São Lucas – UNISL, Afya Porto Velho, Porto Velho, RO, Brasil.; 2 Instituto Vascular e Endovascular de Rondônia – IVER, Porto Velho, RO, Brasil.

**Keywords:** trombose venosa profunda, trombectomia, procedimentos endovasculares

## Abstract

Relata-se o primeiro caso documentado na região Norte do Brasil de trombectomia mecânica percutânea (TMP) em paciente jovem com trombose venosa profunda (TVP) ilíaco-fêmoro-poplítea. O paciente apresentava dor e edema significativo no membro inferior esquerdo, com evidência ecográfica de TVP extensa. Optou-se pelo uso do sistema ClotTriever®, com acesso poplíteo e técnica associada de embolectomia com cateter Fogarty. O procedimento transcorreu sem intercorrências e resultou em rápida recuperação clínica. Após 14 meses de seguimento, o paciente encontrava-se assintomático, com eco-Doppler venoso normal. A TMP mostrou-se segura, eficaz e viável como opção terapêutica inovadora em contexto regional. Este relato reforça o papel da técnica como alternativa promissora para o tratamento de TVPs extensas, especialmente em centros fora de grandes áreas urbanas.

## INTRODUÇÃO

A trombose venosa profunda (TVP) consiste na formação de trombos em veias do sistema venoso profundo, mais comumente nos membros inferiores (80-95% dos casos), podendo causar complicações, como síndrome pós-trombótica (SPT) e embolia pulmonar (EP). Sem tratamento adequado, 5 a 15% dos pacientes com TVP evoluem para óbito por EP; além disso, é estimada uma incidência combinada de TVP e EP de 2 casos por 1.000 pessoas ao ano, com taxas de recorrência de até 25%^1,2^ . Em contrapartida, a incidência de SPT em pacientes com TVP gira em torno de 23% a 60% dos casos, ocorrendo com maior frequência nos 2 anos seguintes ao episódio de TVP^3^ .

A trombectomia mecânica percutânea (TMP) representa uma estratégia inovadora no manejo da TVP, permitindo a remoção do trombo de forma eficaz e minimamente invasiva, sem necessidade de uso associado de trombolíticos. Dessa forma, este relato descreve o primeiro caso de TMP realizado na região Norte do Brasil, evidenciando sua aplicabilidade, eficácia e segurança.

O presente estudo foi avaliado e aprovado pelo Comitê de Ética em Pesquisa (CAAE 85170324.0.0000.0013, parecer nº 7.629.445).

## PARTE I – SITUAÇÃO CLÍNICA

Paciente do sexo masculino, 27 anos de idade, deu entrada com queixa de dor e parestesias no membro inferior esquerdo (MIE) com 2 dias de evolução. Referia hipertensão arterial sistêmica e histórico de gastroplastia com *bypass* há 35 dias. Os exames laboratoriais mostraram leucocitose sem desvio, hemoglobina de 15,5 g/dL, hematócrito de 45,8%, tempo de ativação de protrombina de 45,8 segundos e proteína C reativa elevada (149,9 mg/L).

No exame físico, o paciente encontrava-se estável, mas com dor persistente, edema assimétrico, empastamento, pulsos distais diminuídos e cianose não fixa dos pododáctilos à esquerda (Figura 1). A ultrassonografia com Doppler venoso evidenciou TVP envolvendo as veias ilíaca externa, femoral comum, femoral profunda, femoral, poplítea e gastrocnêmias mediais do MIE.

**Figura 1:** Paciente com edema assimétrico extenso e empastamento no membro inferior esquerdo (MIE), compatível com trombose venosa profunda (TVP).

## PARTE II – O QUE FOI FEITO

O procedimento indicado foi TMP com o sistema ClotTriever® (Inari Medical, Inc., EUA), abrangendo os segmentos cavo-ilíaco e fêmoro-poplíteo do MIE. A intervenção foi realizada sob anestesia local e sedação. Com o paciente em decúbito dorsal e MIE fletido, procedeu-se à punção ecoguiada da veia poplítea esquerda e inserção de introdutor 6Fr. A flebografia inicial confirmou TVP desde a veia poplítea até a veia cava inferior (Figura 2).

**Figura 2:** Flebografia realizada após a punção ecoguiada da veia poplítea esquerda e a passagem do introdutor 6Fr. (A) Trombo acometendo o segmento cavo-ilíaco esquerdo; (B) Trombo acometendo o segmento fêmoro-poplíteo esquerdo.

Realizou-se TMP com o sistema ClotTriever®, com remoção de grande volume de trombos recentes (Figura 3). Para alcançar a veia femoral profunda, foi realizada punção da veia femoral comum direita, cateterização da veia femoral profunda esquerda e passagem de introdutor longo 6Fr x 55 cm. A embolectomia com cateter Fogarty nº 4 permitiu o direcionamento do trombo para o ClotTriever®, que foi posicionado na veia ilíaca externa esquerda (Figura 4). A flebografia de controle demonstrou perviedade dos segmentos tratados, ausência de trombos residuais e fluxo contínuo (Figura 5).

**Figura 3:** **(**A) Inserção do cateter na topografia fêmoro-poplíteo; (B) Trombo extenso capturado pela bolsa coletora; (C) Comprimento da área acometida e proporção da bolsa coletora.

**Figura 4:** Radioscopia demonstra o procedimento de remoção de trombo da veia femoral profunda esquerda. (A) Inserido pela veia femoral comum direita, o introdutor longo 6Fr x 55cm foi posicionado na origem da veia femoral profunda esquerda. Pelo introdutor longo, o cateter Fogarty n° 4 foi inserido, posicionado no segmento distal da veia femoral comum esquerda e insuflado com contraste diluído em solução fisiológica; (B) O sistema ClotTriever® foi posicionado aberto no segmento ilíaco esquerdo e o conjunto introdutor-Fogarty foi tracionado até a veia femoral comum.

**Figura 5:** A imagem demonstra flebografia após trombectomia realizada. É possível ver a perviedade das veias tratadas, indicando a remoção eficaz dos trombos previamente identificados. (A) Segmento cavo-ilíaco; (B) Segmento fêmoro-poplíteo.

O paciente foi encaminhado à unidade de terapia intensiva (UTI) e iniciou anticoagulação plena com enoxaparina 100 mg a cada 12 horas, elastocompressão, elevação do membro e deambulação precoce. No dia seguinte, apresentou melhora clínica acentuada, com regressão do edema e ausência de complicações (Figura 6). A angiotomografia de tórax não evidenciou sinais de EP. Após 1 dia na UTI e 2 dias em enfermaria, recebeu alta. Em seguimento ambulatorial após 14 meses do procedimento, encontrava-se assintomático e com ultrassom Doppler venoso profundo normal (Figura 7).

**Figura 6:** Fotografia demonstrando redução do edema no membro afetado no dia seguinte à cirurgia. A melhora do edema sugere restabelecimento do fluxo venoso, sem sinais de complicações pós-operatórias, confirmando a evolução favorável do paciente.

**Figura 7:** Ecografia vascular com Doppler colorido realizada 14 meses após a trombectomia mecânica percutânea. (A) Veia femoral comum esquerda com fluxo venoso preservado e recanalização completa; (B) Doppler espectral da veia femoral comum esquerda, demonstrando fluxo fásico respiratório; (C) Veia femoral superficial esquerda com perviedade mantida e ausência de imagens compatíveis com trombos residuais; (D) Veia poplítea esquerda com fluxo adequado e ausência de refluxo venoso ao Doppler espectral, confirmando recuperação hemodinâmica satisfatória. ESQ = esquerda; JSF = junção safeno-femoral; REF = refluxo; VFC = veia femoral comum; VFS = veia femoral superficial; VP = veia poplítea.

## DISCUSSÃO

A TMP representa uma alternativa eficaz ao tratamento convencional da TVP, especialmente em casos extensos, como o descrito. Ao evitar o uso de trombolíticos, reduz-se o risco de sangramentos e outras complicações^4^ . Além disso, a TMP tem sido associada a um menor tempo de internação, sendo uma resolução clínica rápida e promovendo menor incidência de SPT^5,6^ .

Neste caso, a indicação da TMP se deu pela extensão da TVP e pelo quadro clínico grave. O desfecho favorável sugere a segurança e a efetividade do método, contribuindo para sua validação como abordagem terapêutica especialmente em centros com estrutura de hemodinâmica e expertise endovascular^7^ .

Além da eficácia clínica, a TMP favorece o retorno precoce às atividades habituais, reduzindo a necessidade de internações prolongadas e intervenções adicionais^8,9^ . Diante desse prisma, pacientes submetidos ao procedimento apresentam recuperação funcional mais rápida e menor necessidade de intervenção adicional em comparação com terapias convencionais, como foi o caso do paciente neste relato.

A literatura aponta que pacientes submetidos a TMP tendem a apresentar menor necessidade de UTI e recuperação funcional mais rápida, corroborando os achados deste estudo (Tabela 1). A introdução da técnica na região Norte do Brasil representa um avanço significativo, podendo influenciar a adoção do procedimento em outros centros especializados.

**Tabela 1:** Resultados da literatura que corroboram resultados positivos referentes à TMP.

**Autores**	**Resumo dos resultados**
**Mouawad** **^5^**	Relato de caso: tratamento bem-sucedido da flegmasia cerúlea dolens por TMP em sessão única, restaurando a perviedade venosa e aliviando sintomas.
**Nguyen et al.** **^9^**	Estudo de coorte unicêntrico: manejo ambulatorial da TVP iliofemoral com síndrome de May-Thurner, usando trombectomia, angioplastia e *stent*, teve sucesso técnico e perviedade de 86,7% após 12 meses, quando comparado ao manejo hospitalar.
**Wong et al.** **^10^**	Revisão sistemática: a TMP para TVP iliofemoral aguda teve taxas de perviedade de 75-100%, menores complicações hemorrágicas e menor incidência de SPT. Além disso, as taxas SPT e recorrência de TVP foram menores que 17 e 15%, respectivamente.
**Maldonado et al.** **^11^**	Estudo prospectivo observacional: o sistema ClotTriever® (Inari Medical, Inc., CA, USA) foi eficaz na remoção de trombos em uma única sessão, sem a necessidade de trombolíticos, com taxa de sucesso de remoção completa ou quase completa em mais de 80% dos casos.
**Zia et al.** **^12^**	Revisão retrospectiva unicêntrica: resultados demonstraram que a TMP apresentou taxas semelhantes de reoclusão em relação a outros tratamentos, mas com menos complicações hemorrágicas e sem necessidade de estadia em UTI.
**Abramowitz et al.** **^13^**	Ensaio clínico randomizado: o estudo mostrou que a trombectomia mecânica com o sistema ClotTriever® é segura e eficaz em TVPs agudas, subagudas e crônicas, com melhorias significativas na qualidade de vida e nos sintomas clínicos, sem diferenças notáveis entre os subgrupos.
**Crowner e Marston** **^14^**	Relato de caso: caso de trombectomia com ClotTriever® em TVP aguda mostrou eficácia e segurança em remover trombos sem trombolíticos, resultando em melhora clínica após 6 meses.

TMP = trombectomia mecânica percutânea; SPT = síndrome pós-trombótica; TVP = trombose venosa profunda; UTI = unidade de terapia intensiva.

A implementação da TMP para tratamento de TVP na região Norte do país enfrenta sérias barreiras de acessibilidade, pois diferentemente do AVC isquêmico, que conta com a PORTARIA SCTIE/MS Nº 5^15^ , que, por sua vez, garante cobertura pelo Sistema Único de Saúde (SUS), a TMP para TVP não possui previsão de incorporação ampla, o que demonstra as desigualdades do sistema de saúde.

O alto custo do procedimento exclui pacientes que dependem do SUS ou de planos que não cobrem terapias de alta complexidade, forçando muitos a recorrer ao setor privado, com gastos inviáveis para a maioria das pessoas. Apesar da dimensão do SUS, sua limitação em ofertar tratamentos avançados como a TMP, somada à ausência de protocolos claros e à escassez de recursos em regiões periféricas, agrava a desigualdade, especialmente no Norte do país.

A incorporação da TMP no SUS se justifica não apenas pelo benefício clínico imediato, mas também pela economia global, com redução de complicações crônicas e desigualdades de acesso. Assim, espera-se que a divulgação deste resultado estimule a adoção da TMP em centros especializados, ampliando o acesso a terapias inovadoras e reduzindo a morbimortalidade associada à TVP. Novos estudos são necessários para consolidar sua incorporação aos protocolos nacionais de tratamento.

## CONCLUSÃO

Neste caso, a TMP mostrou-se segura e eficaz no manejo da TVP extensa, com rápida recuperação clínica e ausência de complicações. O presente relato representa o primeiro uso documentado dessa técnica na região Norte do Brasil, destacando seu valor na expansão de abordagens minimamente invasivas em contextos regionais.

## Data Availability

Data sharing does not apply to this article, as no data were generated or analyzed.
